# Reliability of FEV_1_/FEV_6_ to Diagnose Airflow Obstruction Compared with FEV_1_/FVC: The PLATINO Longitudinal Study

**DOI:** 10.1371/journal.pone.0067960

**Published:** 2013-08-01

**Authors:** Rogelio Perez-Padilla, Fernando C. Wehrmeister, Bartolome R. Celli, Maria Victorina Lopez-Varela, Maria Montes de Oca, Adriana Muiño, Carlos Talamo, Jose R. Jardim, Gonzalo Valdivia, Carmen Lisboa, Ana Maria B. Menezes, for the PLATINO Team

**Affiliations:** 1 National Institute of Respiratory Diseases (INER), Mexico City, Mexico; 2 Post-Graduate Program in Epidemiology, Federal University of Pelotas, Pelotas, Brazil; 3 Pulmonary and Critical Care Department, Brigham and Women's Hospital, Harvard Medical School, Boston, Massachusetts, United States of America; 4 Facultad de Medicina, Universidad de la Republica, Montevideo, Uruguay; 5 Facultad de Medicina, Universidad Central de Venezuela, Caracas, Venezuela; 6 Federal University of São Paulo, São Paulo, Brazil; 7 Pontifícia Universidad Católica de Chile, Santiago de Chile, Chile; Clinica Universidad de Navarra, Spain

## Abstract

**QUESTION:**

A 6-second spirometry test is easier than full exhalations. We compared the reliability of the ratio of the Forced expiratory volume in 1 second/Forced expiratory volume in 6 seconds (FEV_1_/FEV_6_) to the ratio of the FEV_1_/Forced vital capacity (FEV_1_/FVC) for the detection of airway obstruction.

**METHODS:**

The PLATINO population-based survey in individuals aged 40 years and over designed to estimate the prevalence of post-Bronchodilator airway obstruction repeated for the same study participants after 5–9 years in three Latin-American cities.

**RESULTS:**

Using the FEV_1_/FVC<Lower limit of normal (LLN) index, COPD prevalence apparently changed from 9.8 to 13.2% in Montevideo, from 9.7 to 6.0% in São Paulo and from 8.5 to 6.6% in Santiago, despite only slight declines in smoking prevalence (from 30.8% to 24.3%). These changes were associated with differences in Forced expiratory time (FET) between the two surveys. In contrast, by using the FEV_1_/FEV_6_ to define airway obstruction, the changes in prevalence were smaller: 9.7 to 10.6% in Montevideo, 8.6 to 9.0% in São Paulo, and 7.5 to 7.9% in Santiago. Changes in the prevalence of COPD with criteria based on FEV_1_/FVC correlated strongly with changes in the FET of the tests (R^2^ 0.92) unlike the prevalence based on a low FEV_1_/FEV_6_ (R^2^ = 0.40).

**CONCLUSION:**

The FEV_1_/FEV_6_ is a more reliable index than FEV_1_/FVC because FVC varies with the duration of the forced exhalation. Reporting FET and FEV_1_/FEV_6_<LLN helps to understand differences in prevalence of COPD obtained from FEV_1_/FVC-derived indices.

## Introduction

Accurate determination of the prevalence of Chronic obstructive pulmonary disease (COPD) is needed so that the allocation of health care resources may have the desired impact. Further, to determine whether plans such as smoking cessation programs or therapy are effective in decreasing COPD incidence and prevalence it is necessary to have reliable spirometric indices that can accurately detect the disease state and its changes.

The ratio of Forced expiratory volume in one second (FEV_1_) to Forced vital capacity (FVC) has been the parameter-of-choice to define the presence of airflow limitation. However, its interpretation has been a matter of intense debate because its ultimate value depends not only on the degree of airflow obstruction but also on the value of the FVC, which in turn is heavily influenced by the duration of the expiratory time. With slow lung emptying, as occurs with aging and especially in individuals with airflow obstruction, FVC is sensitive to the expiratory time: the longer the expiratory time, the larger the FVC and the smaller the FEV_1_/FVC.

The six-second spirometry has been proposed as a simplified alternative to an FVC maneuver [1]–[7]. Indeed, the ratio of the FEV_1_/Forced expiratory volume in 6 seconds (FEV_6_) has been found nearly equivalent to FEV_1_/FVC for the diagnosis of COPD, but the former is simpler, causes less fatigue, and is possibly more specific than FEV_1_/FVC [8]. In addition, several inexpensive electronic devices measure FEV_1_ and FEV_6_, improving the Peak flow measurement and offering truly low-cost spirometry [9]–[11]. However, fewer reference values are available for FEV_6_ and FEV_1_/FEV_6_ compared with the gold standard FVC and FEV_1_/FVC [12]–[15].

The quality of spirometry varies among technicians and among centers participating in a collaborative study; thus, changes in personnel over time may influence repeated measurements of the same individuals. To correct for this, one of the components of spirometry quality is the duration of the expiratory maneuvers. Current American Thoracic Society (ATS)/European Respiratory Society (ERS) quality standards for spirometry [16] define a valid expiration as one lasting at least 6 seconds with an end-of-test volume (EOTV) of <25 mL during the final second. Many modern spirometers perform automatic checks for maneuver acceptability and repeatability and provide messages and quality grades. However the spirometer operator is free to ignore these messages. We hypothesized that if the expiratory flow were low, FVC and consequently FEV_1_/FVC, may differ if the expiration lasts 6, 8, 10, or more seconds especially if emptying has not been complete. Under these circumstances, a 6-second spirometry may be a more stable indicator because individual results are compared at fixed predetermined times.

The purpose of this work was to compare estimates of COPD prevalence based on different indices of airway obstruction across Latin American Project on Pulmonary Obstruction (PLATINO) Study centers and between baseline and follow-up spirometries performed at three of the PLATINO study sites.

## Population and Methods

### Ethics statement

The study protocol was approved by the Ethics Committee on Research, Pontificial Catholic University of Chile School of Medicine, by the Ethics Committee of the Maciel Hospital in Montevideo Uruguay, and by the Ethics Committee on Research of the Federal University of São Paulo/São Paulo Hospital. Study participants provided signed informed consent.

Details of the selection method and the population sample size of the PLATINO baseline have been previously published [17]. Multistage cluster sampling was used to obtain a representative sample of subjects aged 40 years or over from the metropolitan area of each of the following five large Latin American cities: Montevideo; São Paulo; Santiago; Mexico City, and Caracas.

Study questionnaires are available on the Internet (http://www.platino-alat.org) and a detailed description of the PLATINO study completed questionnaires has been published elsewhere [18]–[21].

Spirometry was performed utilizing the portable, battery-operated, ultrasound EasyOne spirometer (ndd Medical Technologies, Zurich, Switzerland). Spirometry tests were performed at baseline and 15 min after the administration of 200 µg of salbutamol post -Bronchodilation (post-BD), with the goal of meeting American Thoracic Society (ATS) acceptability and repeatability criteria [22]. We employed the definition and the severity stratification of airway obstruction proposed by the Global Initiative for Obstructive Lung Disease (GOLD): a ratio of the post-Bronchodilator (post-BD) FEV_1_ over FVC<0.70 [23]. We also applied the Lower limit of normal (LLN) criteria [8], [24], [25], defined as the lower 5th percentile for predicted post-BD FEV_1_/FEV_6_ and FEV_1_/FVC utilizing equations derived from the baseline examination of the healthy, never-smoking subset of our cohort [26].

Quality control of spirometry testing was described previously [27], [28], and procedures included in all centers identical spirometers, homogeneous training of the technicians, and review of all tests carried out by one expert (R-P) with weekly quality reports per technician and participating center. A six-category quality grade was assigned to each test according to the number of acceptable maneuvers and to the repeatability of FEV_1_ and FVC following the ATS criteria. Grade A quality tests had three acceptable maneuvers with the best two FEV_1_ and FVC within 150 mL [16], [22]; grade B was equivalent to the 1994 ATS criteria, with three acceptable maneuvers with the two best FEV_1_ and FVC matching within 200 mL; grade C tests had two or three acceptable maneuvers repeatable within 250 mL; grade D tests had 2–3 acceptable maneuvers with poor repeatability; grade E tests had only one acceptable test; and grade F tests had no acceptable maneuvers.

In three cities (Montevideo, Santiago, and São Paulo), a second survey was performed after approximately 5, 6, and 9 years, respectively, on the same individuals recruited for the first evaluation using the same spirometers and techniques. All technicians were urged to obtain grade A spirometries in both evaluations, but technicians differed among the three cities and there was technician turnover between the baseline and the follow-up.

Descriptive analyses included group comparisons using the Pearson χ^2^ test for nominal variables, the Mann-Whitney *U* test and ordered logistic regression for ordinal variables, and the Wald test for continuous variables. Linear and logistic regression models were employed to evaluate multivariable relationships.

All analyses were performed using a commercially available statistical software package (Stata v10.0) (StataCorp, College Station, TX, USA) with the survey (svy) commands that consider sampling strategy (cities and basic geostatistical areas).

## Results

A total of 2,136 spirometries (2,064 post-BD) were obtained from the 2,201 individuals interviewed in Montevideo, São Paulo, and Santiago and used in the analysis along with 3,021 spirometries (2,942 post-BD) from 3,151 baseline participants. Main anthropometric and post-BD spirometric data, as well as smoking prevalence, history of self-reported asthma, and COPD of these subjects, are described in Table 1. (For the pre-BD spirometry results see Table S1).

**Table 1:** PLATINO study participants' characteristics and post-BD spirometry results by study center.

	Montevideo, Uruguay		São Paulo, Brazil		Santiago, Chile	
	Baseline	Follow-up	Baseline	Follow-up	Baseline	Follow-up
***N***	943	683	1000	612	1208	898
**Age (years)**	60.3 (12.7)	63.5 (12.1)	55.2 (11.3)	62.4 (9.9)	57.0 (12.0)	62.1 (11.0)
**Male**	40.3% (37.2; 43.5)	40.6% (36.9; 44.3)	44.2% (41.1; 47.3)	44.1% (40.2; 48.1)	38.5% (35.7; 41.2)	36.3% (33.2; 39.5)
**Obese**	33.8% (30.8; 36.8)	39.4% (35.7; 43.1)	25.4% (22.7; 28.1)	32.7% (29.0; 36.5)	32.2% (29.5; 34.8)	33.5% (30.4; 36.6)
**Height (cm)**	161.0 (10.1)	160.4 (10.3)	160.1 (9.5)	159.9 (10.3)	159.2 (9.6)	158.9 (9.5)
**Weight (kg)**	73.5 (16.6)	74.8 (15.8)	70.0 (15.7)	72.4 (15.6)	72.2 (14.1)	72.7 (14.4)
**BMI (Kg/m^2^)**	28.3 (5.7)	29.0 (5.4)	27.3 (5.6)	28.5 (8.1)	28.5 (5.0)	28.7 (5.2)
**FEV_1_ (L)**	2.62 (0.82)	2.54 (0.80)	2.68 (0.79)	2.43 (0.72)	2.70 (0.79)	2.52 (0.77)
**FVC (L)**	3.43 (1.02)	3.42 (1.05)	3.44 (0.95)	3.05 (0.84)	3.52 (0.97)	3.27 (0.93)
**FEV_1_/FVC (%)**	76.4 (9.0)	74.8 (9.6)	78.0 (9.2)	79.2 (6.9)	76.6 (8.7)	76.8 (8.1)
**FEV_6_ (L)**	3.30 (0.97)	3.22 (0.96)	3.31 (0.90)	3.02 (0.84)	3.37 (0.93)	3.18 (0.90)
**FEV_1_ (% ** ***pred*** **)**	96.6 (17.6)	101.4 (19.3)	95.1 (18.3)	95.3 (18.9)	98.6 (16.5)	100.6 (18.9)
**FVC (% ** ***pred*** **)**	99.1 (15.5)	103.1 (18.3)	97.7 (17.3)	91.7 (15.9)	102.3 (14.1)	100.2 (16.4)
**FEV_6_ (% ** ***pred*** **)**	99.2 (15.5)	100.9 (16.4)	97.3 (16.3)	93.8 (16.3)	101.7 (14.2)	100.5 (15.7)
**FEV_1_/FEV_6_ (%)**	79.4 (6.9)	78.8 (7.0)	80.7 (7.3)	80.2 (6.6)	79.6 (6.7)	78.9 (6.8)
**Prevalence of current smoking**	28.0% (25.2; 30.9)	22.0% (18.9; 25.1)	23.9% (21.3; 26.6)	14.8% (12.0; 17.7)	38.5% (35.7; 41.2)	32.5% (29.4; 35.6)
**Pack-years of smoking**	15.8 (25.1)	14.5 (22.4)	11.8 (18.7)	12.8 (20.8)	9.4 (14.9)	10.2 (16.9)
**Self-reported asthma** *	14.0% (11.8; 16.2)	15.7% (13.0; 18.5)	10.1% (8.2; 12.0)	10.4% (8.0; 12.9)	20.9% (18.6; 23.2)	19.1% (16.5; 21.6)
**Self-reported asthma** * **with current asthma** **	4.9 (3.5; 6.3)	5.8 (4.1; 7.6)	3.1 (2.0; 4.2)	6.2 (4.3; 8.1)	5.9 (4.5; 7.2)	6.0 (4.5; 7.6)
**Self-reported COPD** *	2.4 (1.5; 3.4)	3.5 (2.2; 4.9)	5.3 (3.9; 6.7)	8.2 (6.0; 10.3)	6.0 (4.6; 7;3)	7.3 (5.6; 9.0)

* Asthma and Chronic obstructive pulmonary disease (COPD) diagnosed previously by a physician.

** Asthma remains present at the survey time.

Post-BD = tests tests performed after bronchodilator use. Prevalences were estimated taking the study design into account. PLATINO = The Latin American Project of Research on Obstructive Lung Disease; SD = Standard deviation; L = Liters; BMI = Body mass index; FEV_1_ = Forced expiratory volume in 1 second; FEV_6_ = Forced expiratory volume in six seconds; FVC = Forced vital capacity. See Text S1 for pre bronchodilator tests.

The prevalence of COPD and post-BD airflow obstruction, by means of several definitions, is described in Table 2, along with spirometry quality criteria including mean Forced expiratory time (FET) and within-test Coefficient of variability (COV) for several spirometry indicators. Spirometry quality varied among the sites during both the baseline and follow-up surveys and decreased during the second survey. The within-test COV for FEV_6_ was lower than that for FVC, and the within-test COV for FEV_1_/FEV_6_ was considerably lower (60%) than that of FEV_1_/FVC both prior to (pre-BD) and after (post-BD) bronchodilator use.

**Table 2:** Prevalence (%) of post-BD airway obstruction and indices of spirometry quality by study site.

	Montevideo, Uruguay		São Paulo, Brazil		Santiago, Chile	
	Baseline	Follow-up	Baseline	Follow-up	Baseline	Follow-up
**FEV_1_/FVC<0.7**	19.5 (17.1; 22.0)	27.5 (24.0; 31.0)	15.7 (13.4; 17.9)	8.5 (6.4; 10.6)	16.2 (14.1; 18.3)	15.2 (12.6; 17.7)
**GOLD stages 2–4**	7.8 (6.0; 9.6)	8.4 (6.7; 10.0)	6.1 (4.7; 7.4)	5.3 (3.4; 7.2)	5.8 (4.4; 7.2)	6.0 (4.4; 7.7)
**FEV_1_/FVC<LLN**	9.8 (7.9; 11.8)	13.2 (10.7; 15.6)	9.7 (7.8; 11.6)	6.0 (4.0; 7.9)	8.5 (7.1; 10.1)	6.6 (5.1; 8.2)
**FEV_1_/FEV_6_<LLN**	9.7 (7.8; 11.7)	10.6 (8.8; 12.5)	8.6 (6.9; 10.3)	9.0 (6.7; 11.2)	7.5 (6.1; 9.0)	7.9 (6.3; 9.6)
**FET [mean (SD)]**	10.4 (3.8)	11.7 (5.1)	9.5 (3.3)	7.2 (1.0)	10.8 (3.4)	8.9 (2.1)
**FET (median, IQR)**	9.7 (7.8; 12.1)	10.3 (8.2; 13.9)	9.3 (7.2; 10.8)	7.0 (6.6; 7.7)	10.2 (8.4; 12.4)	8.8 (7.5; 9.9)
**Quality grade A,B,C post-BD (% of tests)**	97.4 (96.3; 98.5)	93.9 (91.6; 96.9)	96.2 (94.8, 97.5)	92.9 (90.9; 95.0)	98.9 (98.2; 99.5)	95.3 (93.7 96.9)
**Quality grade A, post-BD (% of tests)**	93.0 (91.2; 94.3)	84.0 (80.8; 87.2)	83.1 (80.4; 85.6)	68.8 (65.3; 72.3)	92.8 (91.2; 94.4)	81.3 (78.2; 84.3)
**FET≥6 s (% of tests)**	95.1 (93.7; 96.6)	95.2 (93.6; 96.8)	90.5 (88.6; 92.4)	96.9 (95.5; 98.4)	98.9 (98.4; 99.5)	96.3 (95.1; 97.6)
**COV for FVC, mean (SD)**	1.4 (1.6)	2.0 (2.0)	1.9 (2.3)	1.7 (2.9)	1.3 (1.5)	1.4 (3.9)
**COV for FEV_6_ ,mean (SD)**	1.4 (1.6)	1.6 (1.7)	1.7 (2.1)	1.6 (2.8)	1.3 (1.5)	1.2 (1.1)
**COV for FEV_1_/FVC ,mean (SD)**	1.0 (1.3)	1.3 (1.5)	1.2 (1.6)	1.0 (1.4)	0.9 (0.8)	1.0 (1.6)
**COV for FEV_1_/FEV_6_ mean (SD)**	0.6 (0.9)	0.7 (0.8)	0.7 (0.9)	0.7 (0.9)	0.5 (0.5)	0.6 (2.3)

FET = Forced expiratory time; Grade A = fulfilling quality criteria by American Thoracic Society/European Respiratory Society (ATS-ERS 2005), three acceptable maneuvers with Forced expiratory volume in one second (FEV)_1_ and Forced vital capacity (FVC) reproducible to 150 mL; Grade A,B,C = Adequate quality tests by (ATS-ERS 2005): two or three acceptable maneuvers with FEV_1_ and FVC reproducible to 250 mL. COV = within-test coefficient of variability. LLN = Lower limit of normal according to PLATINO without bronchodilator reference values. See Text S1 for pre-BD results.

**Figure 1:**
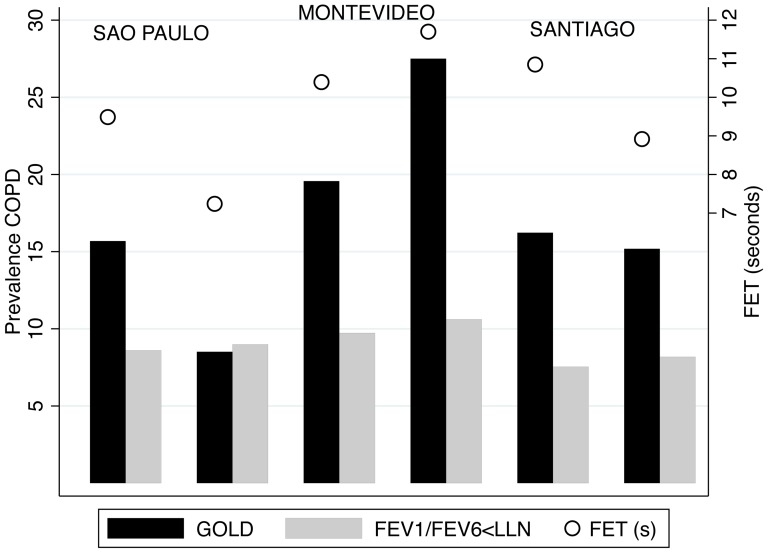
Prevalence of Chronic obstructive lung disease (COPD) by Global Initiative for Chronic Obstructive Lung Disease (GOLD) criteria (black bars) based on Forced expiratory volume in one second (FEV_1_)/Forced vital capacity (FVC)<0.7, and by FEV_1_/FEV_6_<Lower limit of normal (LNN) (<5th percentile for FEV_1_/FEV_6_, gray bars) in the three cities during baseline and follow-up. In addition, mean Forced expiratory time (FET) is plotted (right scale, empty circles) for the post-Bronchodilator tests (post-BD). Prevalence of COPD by FEV_1_/FVC criteria (GOLD) varies directly with Forced expiratory time (FET), unlike prevalence based on FEV_1_/FEV_6_. Prevalences based on a FEV_1_/FVC<LLN also depend to a great extent on FET (not shown).

The prevalence of COPD among cities participating in the baseline and longitudinal PLATINO evaluation is shown in Table 2. Using definitions based solely on FEV_1_/FVC there was an apparently large increase in prevalence in Montevideo, whereas the prevalence appeared to be lower in São Paulo and Santiago. Prevalence correlated well with a longer average Forced expiratory time (FET) in the tests, with shortest mean FET in Sao Paulo and longest in Montevideo (Table 2) (Figure 1).

Analysis restricted to patients in GOLD stages 2–4 shows that prevalence in the first and second evaluations were very similar, whereas employing a FEV_1_/FEV_6_<LLN definition, prevalence increased slightly in all three cities and the measurement was independent of mean FET (see Figure 1).

Data analysis by technician and by city shows that the mean FET explained a higher percentage of variability of FEV_1_/FVC than that of FEV_1_/FEV_6_ (complete analysis at baseline, by city and by technician is provided in Tables S2 and S3).

### Variability in COPD Prevalence Among Cities

Using definitions based on FEV_1_/FVC<0.7 leads to considerable variations in COPD prevalence among cities in the baseline study (from 15.7% in São Paulo to 19.5% in Montevideo) and more marked in the follow-up (from 8.5% in São Paulo to 27.5% in Montevideo), similar to what was observed with the FEV_1_/FVC<LLN (from 8.5% in Santiago to 9.8% in Montevideo at baseline and from 6.0% in São Paulo to 13.2% in Montevideo during follow-up). Variations in prevalence were lower utilizing a GOLD stages 2–4 definition (from 5.8% in Santiago to 7.8% in Montevideo at baseline and from 5.3% in São Paulo to 8.4% in Montevideo during follow-up). The differences were even narrower with the use of the FEV_1_/FEV_6_<LLN (from 7.5% in Santiago to 9.7% in Montevideo at baseline and from 7.9% in Santiago to 10.6% in Montevideo) (see Table 2).

## Discussion

The PLATINO longitudinal study on the prevalence of COPD in three Latin American cities possessed two important findings: first, it showed that the use of a ratio that fixes the time of exhalation (FEV_1_/FEV_6_) is more robust in providing comparisons on COPD prevalence than the fixed FEV_1_/FVC and the FEV_1_/FVC using LLN. This is due to the decrease in the variability of results introduced by differences in the duration of expiration after the minimal value of 6 seconds recommended by the ATS/ERS guidelines is reached. Second, using the FEV_1_/FEV_6_ criteria there is a stabilization or slight increase in the prevalence of airflow obstruction in the three cities surveyed.

**Figure 2:**
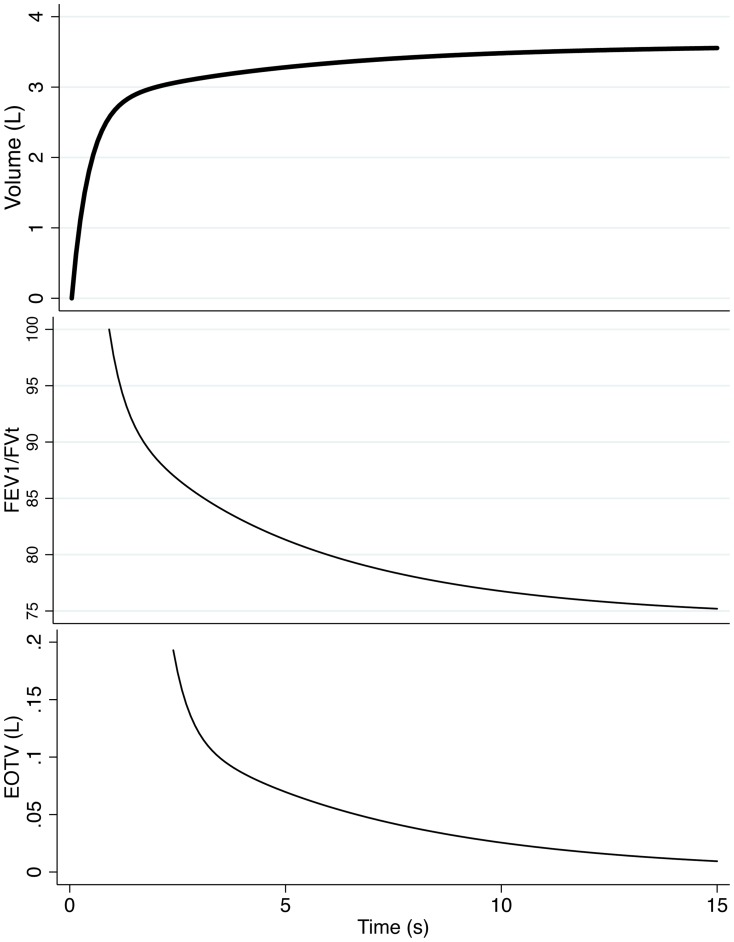
Volume exhaled as a function of expiratory time (upper graph), FEV_1_/FEV_t_ (middle graph) and End-of-test volume EOTV (lower graph). The spirogram is a composite of the PLATINO baseline study based on a bi-exponential fit on individual data (see Text S1). As expiration is prolonged, EOTV decreases as well as the observed FEV_1_/FVC which increases the likelihood of diagnosing airflow obstruction by any FEV_1_/FVC-based criteria.

In this longitudinal population study using the same cohort of subjects, we found conflicting prevalence data using criteria derived from FEV_1_/FVC, the gold standard, with that derived from FEV_1_/FEV_6_. Prevalence of COPD based on the fixed ratio (FEV_1_/FVC<0.7) criteria was higher than that estimated by the FEV_1_/FVC<LLN or the FEV_1_/FEV_6_<LLN criteria, but in addition, during the follow-up survey, this increased significantly in Montevideo (from 19.5 to 27.5%), whereas prevalence in São Paulo apparently decreased from 15.7 to 8.5% and in Santiago, from 16.2 to 15.2%. In a relatively short time, such as that between the two evaluations in the PLATINO Study, the changes in COPD prevalence based on the fixed ratio criteria were unusual and unlikely, and even more so on observing a significant decrease in smoking prevalence in both cities (Montevideo and São Paulo). This discrepancy in results persisted even when using the more specific FEV_1_/FVC<LLN criteria (see Table 2). As such large changes in the prevalence of a chronic disease are unlikely; heterogeneity in mean FET across participating cities and along time by varying spirometric technique may have caused a spurious change in the recorded prevalence, despite the overall good quality of the spirometric tests (quality grades A,B,C, see Table 2). Several lines of evidence support this explanation: first, by restriction of the analysis to GOLD stages 2–4, a criterion requiring not only a low FEV_1_/FVC but also a low FEV_1_. As observed in Table 2, the results did not exhibit the same pattern, but rather tended to decrease the differences observed in the larger sample. When the analysis was repeated using the FEV_1_/FEV_6_<LLN criteria for airflow obstruction the prevalence in Montevideo increased from 9.7% to 10.6%, in Santiago from 7.5% to 7.9%, and from 8.6% to 9.0% in São Paulo. In addition, there were no significant changes in self-reported asthma or COPD, which could have accounted for the huge variations in airflow obstruction as estimated by the fixed ratio criteria and FEV_1_/FVC<LLN (see Table 1). The second evidence is that the rise in prevalence in Montevideo was associated with a significantly longer mean FET in that city, and the decrease in prevalence in Santiago and São Paulo, with a decrease in the FET. A healthy and young lung empties quickly, and in children, the vital capacity is usually expelled in <3 sec. On the other hand, in older individuals and especially in patients with airflow obstruction, complete emptying takes longer and cannot be achieved in a reasonable expiratory time. Therefore, although a longer FET may be the consequence of airflow obstruction, in the same individuals if FET is shorter, FVC would be underestimated, and because airflow obstruction is usually defined by a low FEV_1_/FVC, obstruction may even disappear spuriously. In the same individuals, if the FET is prolonged due to increased encouragement by technicians during testing, FVC will increase; thus. FEV_1_/FVC would decrease, leading to more individuals with “airflow obstruction” (see Figure 2).

Further support for the use of the FEV_1_/FEV_6_ to harmonize results that could be confounded by technical differences is provided by the within-test COV for FEV_1_/FEV_6_ and FEV_6_. These were lower than those for FEV_1_/FVC and FVC, allowing detection of smaller changes that are important in follow-up studies. FEV_1_/FVC and FVC are more influenced by the FET than FEV_1_/FEV_6_ and FEV_6_, not only at the individual level but also when the analysis is extended to individual technicians and to one city (see Table R3). In fact, the variability of the mean FEV_1_/FVC at the city level, critical for estimating prevalence, depends much more on FET than the FEV_1_/FEV_6_.

In the first PLATINO evaluation in 93.9% of the post-BD tests, FVC was larger than FEV_6_, (at follow-up, similar numbers were 96.5%), demonstrating slow emptying due to age and disease. In other words, with an older population, variations in mean FET among technicians and cities, as expected even with good quality control, would produce spurious variations in airflow obstruction prevalence if based on FEV_1_/FVC to a much greater extent than if based on FEV_1_/FEV_6_. Current spirometric end-of testing criteria require a minimum 6-sec expiration, a <25-mL change in volume in the last second or incapacity to exhale further [16]. Even complying with these criteria, thus having a test of good quality, a different FVC would result if expiration were to last for 8 or 11 or more seconds, as observed in Montevideo compared with São Paulo. It is more practical and technically easier and more reliable to compare volume at a fixed expiratory time, as in the 6-sec spirometry.

Individuals with discrepant spirometric airflow obstruction diagnosis, that is having a low FEV_1_/FVC but normal FEV_1_/FEV_6_, often have a high FVC (>120% of predicted) without a low FEV_1_, unlikely those with a low FEV_1_/FEV_6_ and normal FEV_1_/FVC (see Table S4). This suggests a higher false-positive rate of COPD by low FEV_1_/FVC than by low FEV_1_/FEV_6_.

As shown previously, a clinical diagnosis of COPD has a low sensitivity (20%) and a high rate (67%) of false positives compared to spirometric diagnosis (FEV_1_/FEV_6_<LLN), (see Table S5)

An important finding of this study is the relative stabilization of COPD prevalence in the three cities that were re-surveyed. There was a small increase of 0.9% in Montevideo, of 0.4% in Santiago de Chile, and in São Paulo. These results from the same cohort studied at baseline are likely to be true because although a number of patients with severe airflow obstruction died, survivors were now 5–9 years older and one quarter of these had continued to smoke, likely explaining the observed mild increase in prevalence. To our knowledge, this is the first longitudinal study evaluating the prevalence of COPD in a population of stable subjects and the results are interesting in that there appears to be a stabilization in COPD prevalence. On the other hand, these data also suggests that there continues to be a problem of highly prevalent airflow obstruction and that we must increase our efforts to control all of the factors leading to its genesis.

It is now clear that different criteria for confirmation of airflow obstruction lead to varying prevalences of COPD in cross-sectional studies [8], [25]. In addition, it is clear that GOLD stages 1–4 overestimate the real prevalence of COPD, and it is currently common to report GOLD stages 2–4 or to utilize <LLN criteria as a more specific alternative [8], [25]. However, criteria for airflow obstruction may exert an even greater impact on prevalence in longitudinal evaluations, as we show in this study. According to our data, FEV_1_/FEV_6_ is a better indicator of airflow obstruction, likely due to comparing volumes at fixed times of the expiratory maneuver and avoiding inconsistencies due to changes in the quality of the spirometries and especially in forced expiratory time across different technicians, centers, or along time. The BOLD and PLATINO studies have found a significant variation in the COPD prevalence in different cities that is even >3-fold, based on GOLD stages 1–4 criteria [29], [30], and part of this variation may be due to changes in the quality of spirometries across centers and especially to variations in mean expiratory time. Re-evaluation of the published prevalences using FEV_1_/FEV_6_-based definition or the even more restrictive definition requesting both a FEV_1_/FEV_6_<LLN and a FEV_1_<LLN [25] may result in a harmonization of these seemingly differences in prevalence. In other words, reporting the prevalence of FEV_1_/FEV_6_<LLN helps to better understand differences in COPD prevalence obtained from FEV_1_/FVC-derived indices, especially if mean FET is also reported, in addition to the percentage of spirometric tests fulfilling current ATS-ERS criteria (see also Table S4).

In summary, this longitudinal study shows that the FEV_1_/FEV_6_ is a more robust tool to evaluate differences in airflow obstruction prevalence across sites than FEV_1_/FVC, which has been favored to date and which uses either the <0.7 or LLN criteria. Employing this ratio, the prevalence of COPD appears to have increased slightly over the last 5–9 years in the three cities surveyed. Efforts to control the high prevalence of COPD require re-doubling efforts if we are to decrease the human and economic cost of this disease.

## Supporting Information

Text S1 Simulation of spirometry with expiration of different durations to observe the impact on prevalence of COPD. (DOC)

Table S1 PLATINO participants' characteristics and pre-BD spirometry results and quality by study center. Pre-BD = pre-Bronchodilation; FET = Forced expiratory time; Grade A = fulfilling quality criteria by American Thoracic Society/European Respiratory Society (ATS-ERS 2005), three acceptable maneuvers with Forced expiratory volume (FEV)_1_ and Forced vital capacity (FVC) reproducible to 150 mL; COV = Co-efficient of intra-test variability ; LLN = Lower limit of normal; IQR = interquartile range; *pred* = predicted; SD = Standard deviation. (DOC)

Table S2 Coefficient of determination (*R* ^2^ in percentage) from unadjusted and adjusted multiple regression models of FEV_1_/FVC and FEV_1_/FEV_6_ at baseline and follow-up. The PLATINO Study. Note: Adjusted analysis was performed including variables according their appearance in the Table. The smallest *p* value was considered to determine this sequence. FVC = Forced vital capacity; FEV_1_ = Forced expiratory volume in 1 second; FEV_6_ = Forced expiratory volume in 6 seconds. PLATINO = The Latin American Project of Research on Pulmonary Obstruction. (DOC)

Table S3 Mean variability (adjusted *R* ^2^) explained by mean Forced expiratory time (FET) after bronchodilator use. Values obtained by multiple regression. Mean values after bronchodilator use (post-BD). Data by technicians was obtained summarizing mean Forced expiratory volume (FEV)_1_/Forced vital capacity (FVC), FEV_1_/FEV_6_ and Forced expiratory time (FET) by technician. Similarly conducted by city. (DOC)

Table S4 Characteristics of individuals with discordant diagnosis of post-bronchodilator airflow obstruction by FEV_1_/FVC<LLN and by FEV_1_/FEV_6_<LLN criteria. 95%CI = 95% confidence interval. >10py = % of individuals with smoking >10 pack-years. High-risk COPD = >10py or physician's diagnosed asthma, or physician's diagnosed COPD. LLN = Lower limit of normal according to PLATINO reference values. About one half of individuals with low FEV_1_/FVC and normal FEV_1_/FEV_6_ had a high FVC, therefore questionable airflow obstruction, a position sustained even more so by the scarcity of individuals with low FEV_1_ (4/52 and 2/29). On the other hand, out of the individuals with low FEV_1_/FEV_6_ and a normal FEV_1_/FVC only one had a “high” FEV_6_ with a considerable proportion of individuals with low FEV_1_ (9/29 and 11/42) making more likely the presence of airflow obstruction. There likely were more false positives in low FEV_1_/FVC (because high FVC without a low FEV_1_ is common) than in low FEV_1_/FEV_6_. Individuals in the low FEV_1_ category tend to smoke more than those on the high FVC or FEV_6_ category. One cause of high FVC is zero flow errors in the EasyOne spirometer (prolonging FET after the subject stops exhalation) which falsely increases the measured FVC, falsely reduces FEV_1_/FVC, and causes false-positive interpretations for COPD). Zero-flow errors are generated by moving the mouthpiece during the time when zero flow is determined. These misclassifications are minimized using only the first six seconds of the exhalation (replacing FVC with FEV_6_). (DOC)

Table S5 False-positive and false-negative rates of self-reported COPD compared to two gold standards based on a post-Bronchodilator (post-BD) FEV_1_/FEV_6_<LLN. * Self-reported COPD is a physician's diagnosis of COPD, emphysema or chronic bronchitis reported by the individual. %P is the spirometric value expressed as percentage of predicted by PLATINO reference values. First definition: First evaluation, sensitivity of clinical diagnosis of COPD 0.17, and specificity 0.97; second evaluation, sensitivity 0.23, and specificity 0.95. Second definition: First evaluation, sensitivity of clinical diagnosis of COPD 0.26, and specificity 0.97; second evaluation sensitivity 0.34, and specificity 0.97. Third definition: First evaluation, sensitivity of clinical diagnosis of COPD 0.31, and specificity 0.99; second evaluation sensitivity 0.48, and specificity 0.99. (DOC)
