# Supplementary material for: Reliability of FEV1/FEV6 to Diagnose Airflow Obstruction Compared with FEV1/FVC: The PLATINO Longitudinal Study
Source: PLoS One. 2013 Aug 1;8(8):e67960. doi: 10.1371/journal.pone.0067960 (PMC3731337; doi:10.1371/journal.pone.0067960)
Supplement: Text S1 — (DOC) [file pone.0067960.s001.doc]

**SUPPORTING INFORMATION TEXT**

**Reliability of the Diagnosis of Airflow Obstruction Using FEV_1_/FEV_6_ Compared with FEV_1_/FVC: The Longitudinal PLATINO Study.**

Rogelio Perez- Padilla, Fernando C. Wehrmeister, Bartolome R. Celli, Maria Victorina Lopez Varela, Maria Montes de Oca, Adriana Muiño, Carlos Talamo, Jose Roberto B. Jardim, Gonzalo Valdivia, Carmen Lisboa, Ana Maria B. Menezes, for the PLATINO Team*

**Simulation of spirometry with expiration of different durations to observe the impact on prevalence of COPD.**

Individual spirometry data in the PLATINO baseline study (FEV_1_, FEV_6_ and FVC) was fit to a bi-exponential model of the form:

Volume=Vfast*(1-e^-time/0.4^)+ (Vslow*(1-e^-time/5.0^)

where time is expiratory time in seconds, Vfast represents a rapid-emptying compartment with a time constant of 0.4 seconds, whereas Vslow is a slowly emptying compartment with a time constant of 5 seconds. Volume is the expired volume. With two exponentials, nearly perfect fit can be obtained (R^2^>0.999) with the very limited available data: 4 points (time= 0 with volume= 0, time= 1 with volume=FEV_1_, time =6 with volume=FEV_6_ and time =FET with volume=FVC). The older and more obstructed the individual the higher the volume of slow units. The time constants 0.4 and 5 s, were previously determined to fit well spirometric data and time constants along with percentage of slow and fast units cannot be estimated directly given that only four points were available.

Concordance between estimated values from exponential models and measured values were good.

| Measurement | Concordance correlation coefficient | Slope | Intercept |
| --- | --- | --- | --- |
| FVC post BD, baseline | 1.0 | 0.997 | 0.004 |
| FEV_1_ post BD baseline | 1.0 | 0.998 | 0.004 |
| FEV_6_ post BD baseline | 0.999 | 0.994 | 0.013 |

* Estimated with FET as time. Similar concordance values were found for the follow-up study. BD=Bronchodilation

Once fit, for each individual, expired volume can be estimated changing the expiratory time, including usually measured variables such as FEV_1_ and FEV_6_ with good concordance with measured values (see Table R1) but also unmeasured volumes such as FEV_11_, FEV_12_ and from this the proper ratios simulating a shorter although valid expiration: FEV_1_/FEV_6_, FEV_1_/FEV_7_ or a longer expiration FEV_1_/FEV_11_ and FEV_1_/FEV_12_ and from this the prevalence of airflow obstruction in each city assuming changes in the expiratory time, such as those occurring by different technicians forcing expirations of different durations, can be calculated on comparing to reference values for FEV_1_/FVC.

| Expiratory time (t) | FEV_1_/FEV_t_ | SD | FEV_1/_FVC<LLN | FEV_1/_FVC<0.7 (GOLD) |
| --- | --- | --- | --- | --- |
| 6 | 79.7 | 6.9 | 0.04 | 0.08 |
| 7 | 78.6 | 7.3 | 0.05 | 0.10 |
| 9 | 77.1 | 7.9 | 0.07 | 0.14 |
| 10 | 76.6 | 8.0 | 0.08 | 0.16 |
| 11 | 76.1 | 8.2 | 0.09 | 0.17 |
| 12 | 75.8 | 8.3 | 0.10 | 0.19 |

*Estimated from baseline spirometry data. LLN according to PLATINO reference values.

Long expiratory times increases the prevalence of airflow obstruction whether by the GOLD criteria or by the FEV_1_/FVC<LLN criteria. On the other hand, it is easy to see that FEV_1_/FEV_6_ will not be affected by the duration of expiration, in fact duration is fixed to 6 seconds.

In multiple regression models using the estimated prevalence values as a function of expiratory time (utilizing <LLN for FEV_1_/FVC criteria), prevalence increases on average by 0.95% per second of expiratory prolongation. Including an indicator variable per city, São Paulo had on average 1.8% less prevalence, and Santiago had 1.9% less prevalence than Montevideo.

By the GOLD criteria, on average, prevalence increases 1.9% per second of expiratory prolongation, with São Paulo having 6.3% less prevalence and Santiago 4.9% less prevalence than Montevideo.

Finally, with the summary exponential data (see below), for the PLATINO baseline study shown in Table R2, an average spirometry was plotted including volume changes, how FEV_1_/FEVt ratio decreases with time and how the volume expelled in the previous second (EOTV) decreases with expired time. EOTV reached 25 mL, the current standard, only after 10 seconds of expiration with this composite curve representing the average characteristics of baseline spirometries.

MAIN PARAMETERS OF THE EXPONENTIAL MODELS

| Variable | N | Mean | SD | Min | Max |
| --- | --- | --- | --- | --- | --- |
| Baseline study  One compartment model |  |  |  |  |  |
| Vmax (L) | 2,799 | 3.43 | 0.95 | 0.90 | 7.07 |
| Time constant (s) | 2,799 | 0.69 | 0.30 | 0.36 | 5.99 |
| R^2^ | 2,799 | 0.998 | 0.003 | 0.907 | 1 |
| Two-compartment models, baseline study |  |  |  |  |  |
| Vfast (L) | 2,799 | 2.749 | 0.86 | 0.03 | 5.87 |
| Vslow (L) | 2,799 | 0.859 | 0.49 | 0.0 | 4.24 |
| R^2^ | 2,799 | 0.999 | 0.00 | 0.96 | 1 |
| One-compartment, follow-up |  |  |  |  |  |
| Vmax (L) | 1,962 | 3.23 | 0.92 | 0.82 | 6.61 |
| Time constant (s) | 1,962 | 0.67 | 0.229 | 0.37 | 4.10 |
| R^2^ | 1,962 | 0.998 | 0.003 | 0.95 | 1 |
| Two-compartment model, follow-up |  |  |  |  |  |
| Vfast (L) | 1,962 | 2.57 | 0.82 | 0.245 | 5.80 |
| Vslow (L) | 1,962 | 0.85 | 0.47 | 0.00 | 3.76 |
| R^2^ | 1,962 | 0.999 | 0.00 | 0.988 | 1 |

All were performed in post Bronchodilator (post-BD) tests and if FET>= 6 seconds. N = Number of models conducted. All two-compartment models at baseline and follow-up had a R^2^>0.999, as well as 75% of one-compartment models at baseline and 85% of one-compartment models at follow-up. Vfast and Vslow are best-fit volumes of putative fast emptying (time constant of 0.4 s) and slow emptying (time constant of 5 s) lung compartments, explaining the characteristics of the spirometric volume-time curve. The older and more obstructed the individual, the higher the percentage of lung volume in the slow compartment
