# Supplementary material for: Reliability of FEV1/FEV6 to Diagnose Airflow Obstruction Compared with FEV1/FVC: The PLATINO Longitudinal Study
Source: PLoS One. 2013 Aug 1;8(8):e67960. doi: 10.1371/journal.pone.0067960 (PMC3731337; doi:10.1371/journal.pone.0067960)
Supplement: Table S1 — (DOC) [file pone.0067960.s002.doc]

Table S1. PLATINO participants’ characteristics and pre-BD spirometry results and quality by study center.

|  | **Montevideo, Uruguay** | | **São Paulo, Brazil** | | **Santiago, Chile** | |
| --- | --- | --- | --- | --- | --- | --- |
|  | **Baseline** | **Follow-up** | **Baseline** | **Follow-up** | **Baseline** | **Follow-up** |
| ***N*** | 943 | 683 | 1000 | 612 | 1208 | 898 |
| **FEV_1_ (L)** | 2.51 (0.82) | 2.45 (0.80) | 2.57 (0.79) | 2.33 (0.75) | 2.60 (0.80) | 2.43 (0.76) |
| **FVC (L)** | 3.44 (1.05) | 3.43 (1.07) | 3.41 (1.00) | 2.97 (0.88) | 3.55 (1.01) | 3.22 (0.94) |
| **FEV_1_/FVC (%)** | 72.9 (9.1) | 71.8 (9.5) | 75.7 (9.4) | 78.1 (7.5) | 73.0 (8.6) | 75.1 (8.0) |
| **FEV_6_ (L)** | 3.25 (0.99) | 3.18 (0.97) | 3.26 (0.95) | 2.93 (0.88) | 3.34 (0.96) | 3.12 (0.91) |
| **FEV_1_ (% pred)** | 95.3 (19.1) | 97.7 (19.9) | 94.5 (19.3) | 92.2 (20.2) | 98.3 (18.2) | 97.1 (19.5) |
| **FVC (% *pred*)** | 99.9 (16.7) | 103.4 (19.1) | 97.5 (18.7) | 89.8 (17.1) | 104.1 (16.1) | 98.6 (16.9) |
| **FEV_6_ (% *pred*)** | 97.6 (16.5) | 99.6 (17.1) | 96.1 (17.8) | 91.7 (17.7) | 100.9 (15.9) | 98.5 (16.5) |
| **FEV_1_/FEV_6_ (%)** | 77.0 (7.0) | 76.7 (6.9) | 78.7 (7.6) | 79.0 (7.2) | 77.5 (6.7) | 77.5 (6.8) |
| **FET [mean (SD)]** | 11.9 (4.2) | 13.0 (4.9) | 9.8 (3.4) | 7.1 (1.4) | 12.6 (3.9) | 9.0 (2.2) |
| **FET (median, IQR)** | 11.4 (9.1; 13.9) | 12.2 (9.6; 15.5) | 9.7 (7.9; 11.1) | 7.1 (6.6; 7.7) | 12.2 (9.9; 14.7) | 8.9 (7.5; 9.9) |
| **Quality grade A (% of tests)** | 99.1 (98.5; 99.7) | 94.0 (92.2; 95.8) | 88.8 (86.8; 90.8) | 82.5 (79.4; 85.6) | 94.2 (92.9; 95.5) | 97.7 (96.7; 98.7) |
| **FET ≥6s (% of tests)** | 95.0 (93.6 (96.5) | 96.5 (95.1; 97.9) | 90.3 (88.5; 92.2) | 93.7 (91.8; 95.6) | 98.0 (97.2; 98.8) | 96.4 (95.1; 97.6) |
| **Within-test COV for FVC, mean (SD)** | 1.4 (1.2) | 1.9 (2.1) | 2.3 (4.1) | 2.1 (5.0) | 1.6 (2.5) | 1.2 (1.3) |
| **Within-test COV for FEV_6_ , mean (SD)** | 1.5 (1.4) | 1.7 (2.0) | 2.2 (4.1) | 1.5 (1.8) | 1.5 (2.4) | 1.1 (0.9) |
| **Within-test COV for FEV_1_/FVC , mean (SD)** | 1.1 (1.1) | 1.5 (1.5) | 1.5 (2.7) | 1.4 (2.6) | 1.2 (1.5) | 0.9 (1.2) |
| **Within-test COV for FEV_1_/FEV_6_ , mean(SD)** | 0.7 (0.7) | 0.8 (0.8) | 0.9 (1.7) | 1.0 (2.6) | 0.7 (0.9) | 0.5 (0.7) |

Pre-BD = pre-Bronchodilation; FET = Forced expiratory time; Grade A = fulfilling quality criteria by American Thoracic Society/European Respiratory Society (ATS-ERS 2005), three acceptable maneuvers with Forced expiratory volume (FEV)_1_ and Forced vital capacity (FVC) reproducible to 150 mL; COV = Co-efficient of intra-test variability ; LLN = Lower limit of normal; IQR = interquartile range; *pred*= predicted; SD = Standard deviation.
