# Supplementary material for: Reliability of FEV1/FEV6 to Diagnose Airflow Obstruction Compared with FEV1/FVC: The PLATINO Longitudinal Study
Source: PLoS One. 2013 Aug 1;8(8):e67960. doi: 10.1371/journal.pone.0067960 (PMC3731337; doi:10.1371/journal.pone.0067960)
Supplement: Table S2 — (DOC) [file pone.0067960.s003.doc]

Table S2. Coefficient of determination (*R*² in percentage) from unadjusted and adjusted multiple regression models of FEV_1_/FVC and FEV_1_/FEV_6_ at baseline and follow-up. The PLATINO Study.

|  | **Baseline** | | | | **Follow-up** | | | |
| --- | --- | --- | --- | --- | --- | --- | --- | --- |
|  | **FEV_1_/FVC** | | **FEV_1_/FEV_6_** | | **FEV_1_/FVC** | | **FEV_1_/FEV_6_** | |
| **Variable** | **Unadjusted** | **Adjusted** | **Unadjusted** | **Adjusted** | **Unadjusted** | **Adjusted** | **Unadjusted** | **Adjusted** |
| Expiratory time | 50.5 | 50.5 | 32.3 | 32.3 | 34.0 | 34.0 | 10.7 | 10.7 |
| Age (years) | 11.6 | 55.1 | 10.8 | 37.5 | 8.7 | 40.1 | 8.6 | 17.7 |
| Weight (kg) | 0.2 | 56.5 | 0.6 | 39.1 | 0.9 | 41.1 | 1.5 | 18.9 |
| Height (cm) | 0.7 | 56.5 | 0.4 | 39.1 | 0.1 | 41.4 | 0.1 | 19.7 |
| Schooling (years) | 1.0 | 56.7 | 1.0 | 39.3 | 0.8 | 41.7 | 1.1 | 20.1 |
| Country | 0.5 | 56.9 | 0.5 | 39.4 | 1.6 | 41.8 | 0.6 | 20.2 |
| Skin colour | 0.1 | 57.0 | 0.1 | 39.4 | 0.1 | 42.0 | 0.1 | 20.3 |

Note: Adjusted analysis was performed including variables according their appearance in the Table. The smallest *p* value was considered to determine this sequence. FVC = Forced vital capacity; FEV_1_ = Forced expiratory volume in 1 second; FEV_6_ = Forced expiratory volume in 6 seconds. PLATINO = The Latin American Project of Research on Pulmonary Obstruction;
