# Supplementary material for: Reliability of FEV1/FEV6 to Diagnose Airflow Obstruction Compared with FEV1/FVC: The PLATINO Longitudinal Study
Source: PLoS One. 2013 Aug 1;8(8):e67960. doi: 10.1371/journal.pone.0067960 (PMC3731337; doi:10.1371/journal.pone.0067960)
Supplement: Table S3 — (DOC) [file pone.0067960.s004.doc]

Table S3. Mean variability (adjusted *R*^2^) explained by mean Forced expiratory time (FET) after bronchodilator use

| Level of analysis | Baseline | | Follow-up | |
| --- | --- | --- | --- | --- |
|  | FEV_1_/FVC | FEV_1_/FEV_6_ | FEV_1_/FVC | FEV_1_/FEV_6_ |
| Individual (see Table R1) | 50.5 | 32.3 | 34.0 | 10.7 |
| By technician | 62 | 37 | 38.9 | 5.3 |
| By city | 81.8 | 55.0 | 91.7 | 40.2 |

Values obtained by multiple regression. Mean values after bronchodilator use (post-BD). Data by technicians was obtained summarizing mean Forced expiratory volume (FEV)_1_/Forced vital capacity (FVC), FEV_1_/FEV_6_ and Forced expiratory time (FET) by technician. Similarly conducted by city.
