# Supplementary material for: Reliability of FEV1/FEV6 to Diagnose Airflow Obstruction Compared with FEV1/FVC: The PLATINO Longitudinal Study
Source: PLoS One. 2013 Aug 1;8(8):e67960. doi: 10.1371/journal.pone.0067960 (PMC3731337; doi:10.1371/journal.pone.0067960)
Supplement: Table S4 — (DOC) [file pone.0067960.s005.doc]

Table S4- Characteristics of individuals with discordant diagnosis of post-bronchodilator airflow obstruction by FEV_1_/FVC<LLN and by FEV_1_/FEV_6_<LLN criteria.

| Airflow obstruction criteria | First evaluation | | |  | Second evaluation | | |  |
| --- | --- | --- | --- | --- | --- | --- | --- | --- |
|  | N | % (95% CI) | >10 py (%) | High-risk COPD | N | % (95% CI) | >10 py (%) | High-risk COPD |
| Non-obstructed by FEV_1_/FVC (<LLN) | 2668/2942 | 90.7 | 13.8 | 27.4 | 1887/2064 | 91.4 | 15.9 | 30.4 |
| Obstructed by a low FEV_1_/FVC (<LLN) | 274/2942 | 9.3 (8.3; 10.3) | 17.9 | 47.1 | 177/2064 | 8.6 (7.4-9.7) | 24.1 | 54.6 |
| High FVC (>120% of predicted) | 43/274 | 15.74 | 9.3 | 18.6 | 44/133 | 24.9 | 23.2 | 30.2 |
| Low FEV_1_ (<LLN) | 101/274 | 38.9% | 22.8 | 68.3 | 73/177 | 41.2 | 24.3 | 67.1 |
| Obstructed by a low FEV_1_/FVC but normal FEV_1_/FEV_6_  (FEV_1_/FVC<LLN and FEV_1_/FEV_6_ ≥LLN) | 52/274 | 19.0 (14.1; 23.9) | 21.2 | 32.7 | 31/177 | 17.5 (11.3; 23.7) | 16.1 | 22.6 |
| High FVC (>120% of predicted) | 24/52 | 46.2 (33.5; 58.8) | 12.5 | 16.7 | 15/31 | 43.4 (33.0; 63.8) | 13.3 | 13.3 |
| Low FEV_1_ (<LLN) | 4/52 | 7.7 (0.4-15.0) | 50.0 | 50.0 | 2/29 | 6.5 (0.0-14.8) | 50.0 | 50.0 |
| Non-obstructed by a low FEV_1_/FEV_6_ (<LLN) | 2691/2942 | 91.5 | 13.9 | 27.3 | 1871/2058 | 90.9 | 15.8 | 29.8 |
| Obstructed by a low FEV_1_/FEV_6_ (<LLN) | 251/2942 | 8.5 (7.6; 9.5) | 17.1 | 49.8 | 187/2058 | 9.1 (8.0-10.2) | 25.0 | 60.3 |
| High FEV_6_ (>120% of predicted) | 9/251 | 3.6 | 0 | 22.2 | 24/187 | 12.8 | 26.1 | 43.4 |
| Low FEV_1_ (<LLN) | 106/251 | 42.2 | 22.6 | 67.0 | 81/187 | 43.3 | 28.2 | 70.5 |
| Obstructed by a low FEV_1_/FEV_6_ but with normal FEV_1_/FVC  (FEV_1_/FEV_6_<LLN and FEV_1_/FVC≥LLN) | 29/251 | 11.6 (7.6 15.5) | 17.2 | 44.8 | 42/187 | 22.5 (16.6; 28.3) | 21.4 | 54.8 |
| High FEV_6_ (>120% of predicted) | 1/29 | 3.5 (0; 10.2) | - | - | 1/42 | 2.4 (0; 7.0) | - | - |
| Low FEV_1_ (<LLN) | 9/29 | 31.0 (15.2; 46.9) | 33.3 | 44.4 | 11/42 | 26.2 (12.9; 39.5) | 54.6 | 81.8 |

95%CI = 95% confidence interval. >10py = % of individuals with smoking >10 pack-years. High-risk COPD = >10py or physician´s diagnosed asthma, or physician´s diagnosed COPD. LLN= Lower limit of normal according to PLATINO reference values.

About one half of individuals with low FEV_1_/FVC and normal FEV_1_/FEV_6_ had a high FVC, therefore questionable airflow obstruction, a position sustained even more so by the scarcity of individuals with low FEV_1_ (4/52 and 2/29). On the other hand, out of the individuals with low FEV_1_/FEV_6_ and a normal FEV_1_/FVC only one had a "high" FEV_6_ with a considerable proportion of individuals with low FEV_1_ (9/29 and 11/42) making more likely the presence of airflow obstruction. There likely were more false positives in low FEV_1_/FVC (because high FVC without a low FEV_1_ is common) than in low FEV_1_/FEV_6_. Individuals in the low FEV_1_ category tend to smoke more than those on the high FVC or FEV_6_ category. One cause of high FVC is zero flow errors in the EasyOne spirometer (prolonging FET after the subject stops exhalation) which falsely increases the measured FVC, falsely reduces FEV_1_/FVC, and causes false-positive interpretations for COPD). Zero-flow errors are generated by moving the mouthpiece during the time when zero flow is determined. These misclassifications are minimized using only the first six seconds of the exhalation (replacing FVC with FEV_6_).
