# Supplementary material for: Reliability of FEV1/FEV6 to Diagnose Airflow Obstruction Compared with FEV1/FVC: The PLATINO Longitudinal Study
Source: PLoS One. 2013 Aug 1;8(8):e67960. doi: 10.1371/journal.pone.0067960 (PMC3731337; doi:10.1371/journal.pone.0067960)
Supplement: Table S5 — (DOC) [file pone.0067960.s006.doc]

Table S5- False-positive and false-negative rates of self-reported COPD compared to two gold standards based on a post-Bronchodilator (post-BD) FEV_1_/FEV_6_<LLN.

| Airflow obstruction gold standard | First evaluation (three cities) | | Second evaluation (three cities) | |
| --- | --- | --- | --- | --- |
|  | False negative % | False positive % | False negative % | False positive % |
| Airflow obstruction defined by  (FEV_1_/FEV_6_<LLN) | 208/2804 (7.4) | 93/136 (68.4) | 141/1897 (7.4) | 84/127 (66.1) |
| Airflow obstruction defined by  (FEV_1_/FEV_6_<LLN and FEV_1_ <LLN ) | 78/2804 (2.8) | 108/136 (79.4) | 52/1897 (2.74) | 100/127 (78.7) |
| Clinically significant airflow obstruction  (FEV_1_/FEV_6_<LLN and FEV_1_ <60%P) | 38/2791 (1.4) | 115/132 (87.1) | 21/1870 (1.1) | 104/124 (83.9) |

* Self-reported COPD is a physician´s diagnosis of COPD, emphysema or chronic bronchitis reported by the individual.

%P is the spirometric value expressed as percentage of predicted by PLATINO reference values

First definition: First evaluation, sensitivity of clinical diagnosis of COPD 0.17, and specificity 0.97; second evaluation, sensitivity 0.23, and specificity 0.95. Second definition: First evaluation, sensitivity of clinical diagnosis of COPD 0.26, and specificity 0.97; second evaluation sensitivity 0.34, and specificity 0.97. Third definition: First evaluation, sensitivity of clinical diagnosis of COPD 0.31, and specificity 0.99; second evaluation sensitivity 0.48, and specificity 0.99
